# Myocardial work across different etiologies of right ventricular dysfunction and healthy controls

**DOI:** 10.1007/s10554-023-03038-y

**Published:** 2024-02-02

**Authors:** Kristoffer Berg-Hansen, Nigopan Gopalasingam, Tor Skibsted Clemmensen, Mads Jønsson Andersen, Søren Mellemkjaer, Steen Hvitfeldt Poulsen, Jesper Khedri Jensen, Roni Nielsen

**Affiliations:** 1 Department of Cardiology, Aarhus University Hospital, Palle Juul-Jensens Boulevard 99, Aarhus N, DK-8200 Denmark; 2 Department of Clinical Medicine, Faculty of Health, Aarhus University, Aarhus, Denmark

**Keywords:** Myocardial work, Right ventricle, Pulmonary hypertension, Tricuspid regurgitation

## Abstract

Evaluating right ventricular (RV) function remains a challenge. Recently, novel echocardiographic assessment of RV myocardial work (RVMW) by non-invasive pressure-strain loops was proposed. This enables evaluation of right ventriculoarterial coupling and quantifies RV dyssynchrony and post-systolic shortening. We aimed to assess RVMW in patients with different etiologies of RV dysfunction and healthy controls. We investigated healthy controls (n=17), patients with severe functional tricuspid regurgitation (FTR; n=22), and patients with precapillary pulmonary hypertension (PCPH; n=20). Echocardiography and right heart catheterization were performed to assess 1) RV global constructive work (RVGCW; work needed for systolic myocardial shortening and isovolumic relaxation), 2) RV global wasted work (RVGWW; myocardial shortening following pulmonic valve closure), and 3) RV global work efficiency (RVGWE; describes the relation between RV constructive and wasted work). RVGCW correlated with invasive RV stroke work index (r=0.66,* P*<0.001) and increased in tandem with higher afterload, i.e., was low in healthy controls (454±73 mmHg%), moderate in patients with FTR (687±203 mmHg%), and highest among patients with PCPH (881±255 mmHg%). RVGWE was lower and RVGWW was higher in patients with FTR (86±8% and 91 mmHg% [53-140]) or PCPH (86±10% and 110 mmHg% [66-159]) as compared with healthy controls (96±3% and 10 mmHg%). RVMW by echocardiography provides a promising index of RV function to discriminate between patients with RV volume or pressure overload. The prognostic value of this measure needs to be settled in future studies.

**Graphical abstract:**

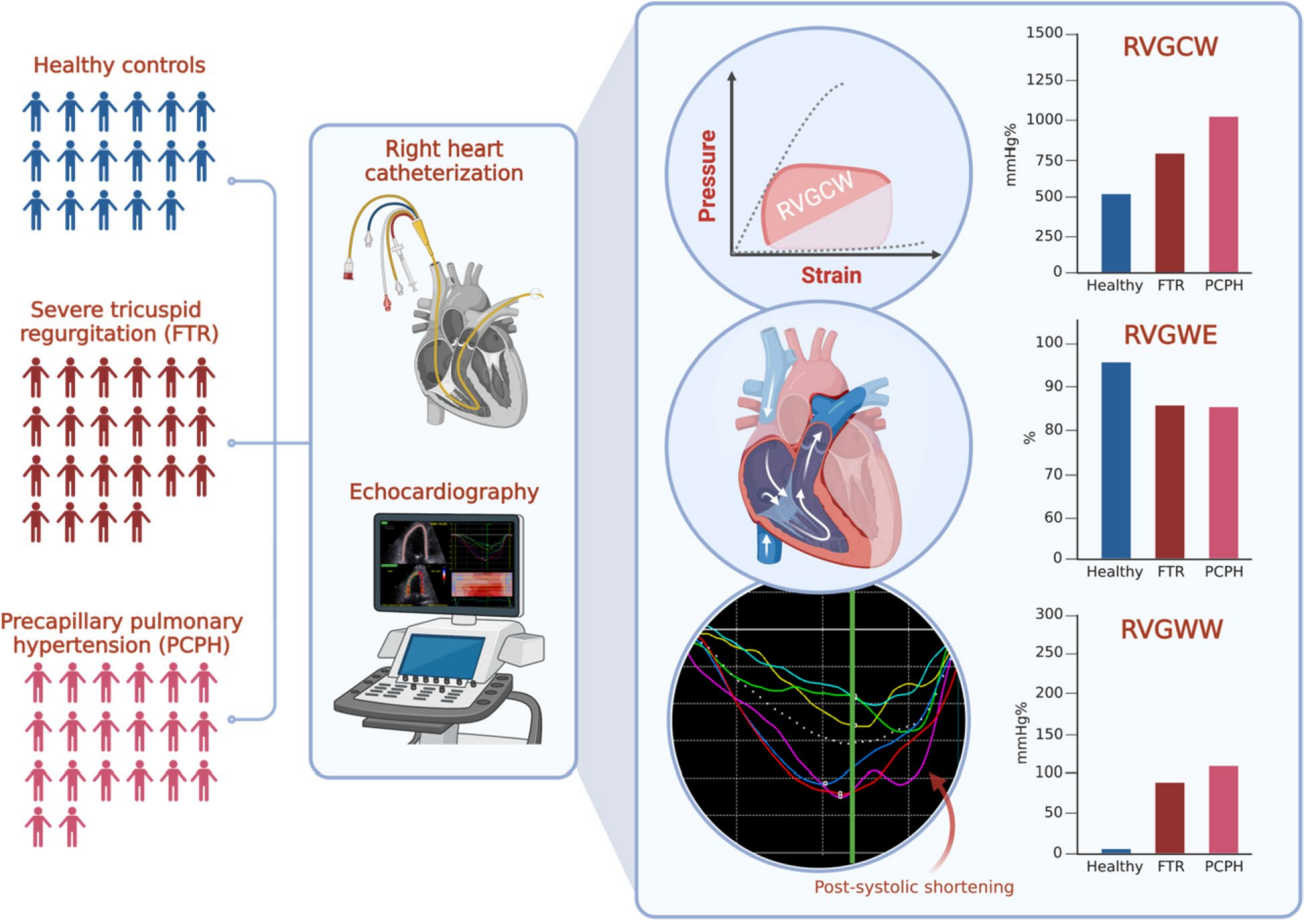
 Combining right heart catheterization and echocardiography, right ventricular (RV) pressure-strain loops were evaluated in healthy controls and in patients with severe functional tricuspid regurgitation (FTR) or precapillary pulmonary hypertension (PCPH). RV global constructive work (RVGCW) entails the work needed for systolic myocardial shortening and isovolumic relaxation; it increased in tandem with higher afterload. RV global wasted work (RVGWW) describes myocardial shortening following pulmonic valve closure and RV global work efficiency (RVGWE) is the ratio between RVGCW and RVGWW. RVGWW was higher and RVGWE was lower in both patient groups with RV hemodynamic overload.

**Supplementary Information:**

The online version contains supplementary material available at 10.1007/s10554-023-03038-y.

## Introduction

Right ventricular (RV) dysfunction is a strong predictor of increased morbidity and mortality in patients with chronic elevated RV pressure or volume overload [1]. However, different etiologies of RV dysfunction may vary significantly in terms of preload, afterload, and geometric remodeling. The transition from a compensated RV to a dysfunctional RV ultimately depends on the capacity of the RV to adapt to the increased RV pressure or volume overload [2]. However, timely identification of RV dysfunction remains challenging. Echocardiographic evaluation of RV function is fundamental in evaluating right heart disease. However, traditional echocardiographic indices of RV performance are subjective to errors of angle, translational motion, and geometric assumptions [3]. Also, echocardiographic parameters fail to assess the ventriculoarterial coupling between the RV and the pulmonary circulation; indeed, RV stroke volume is highly susceptible to changes in afterload [4]. RV global longitudinal strain (GLS) is increasingly recognized as a superior and angle-independent index of RV contractile function [5]. However, RV GLS is also load dependent and prone to error by failing to quantify contractile dyssynchrony and post-systolic shortening [6, 7]. Combining blood pressure and strain to create pressure-strain loops has been introduced as a measure of left ventricular (LV) myocardial work (LVMW) [8]. This method has been applied as a measure of RV function (RV myocardial work; RVMW) in patients with LV systolic dysfunction [9] and patients with precapillary pulmonary hypertension (PCPH) [10]. Yet, there is a paucity of data comparing RVMW between different etiologies of RV dysfunction. In the present study, we aimed to assess pressure-strain loops in patients with PCPH or severe functional tricuspid regurgitation (FTR) and to compare RVMW between these patients and with healthy controls.

## Methods

The present proof-of-concept study is an observational post-hoc investigation of two clinical trials conducted at Aarhus University Hospital between November 2017 and March 2021. Briefly, the aims of study I and II were (I) to examine the hemodynamic characteristics during rest and exercise of patients with FTR [11], and (II) to investigate the hemodynamic effects of ketone body supplements to patients with PCPH [12]. The studies were approved by the local ethics committee and the Danish Data Protection Agency. Participants were included following informed, written consent in accordance with the Helsinki Declaration. The data underlying this article will be shared upon reasonable request to the corresponding author.

Fifty-nine participants were investigated: 17 healthy controls with no cardiopulmonary disease, 22 patients with FTR secondary to clinically stable chronic atrial fibrillation, and 20 patients with PCPH (idiopathic pulmonary arterial hypertension (PAH) or chronic thromboembolic pulmonary hypertension; 10 patients in each group). For patients with PCPH, inclusion criteria were mean pulmonary arterial pressure (mPAP) > 25 mmHg, PVR > 3 Wood units, and pulmonary artery wedge pressure (PAWP) < 15 mmHg on right heart catheterization (RHC) according to the 2015 ESC guidelines on pulmonary hypertension [13]. Common exclusion criteria were LV ejection fraction (LVEF) < 50% and significant left-sided valve disease. Patients with FTR met the criteria for severe tricuspid regurgitation as outlined in the guidelines [14]; these patients were eligible for tricuspid valve repair following study participation.

A 7.5 Fr triple lumen Swan Ganz catheter was advanced through an 8 Fr sheath in the right jugular vein and advanced into the pulmonary artery by fluoroscopy guidance. All pressure recordings were evaluated in the supine position at end-expiration following a minimum of 10 min of rest. Right atrial pressure (RAP), mPAP, and PAWP were measured. Mixed venous saturation (SVO_2_) was recorded. Oxygen consumption (VO_2_) was measured by expired gas analysis (Vyntus CPX, Vyaire medical GMBH, Germany). The difference in arterial-venous O_2_ content (A-VO_2_diff) was calculated as the difference between systemic arterial and SVO_2_ content. Cardiac output (CO) was calculated by direct Fick’s method (CO = VO_2_/A-VO_2_diff) for patients with FTR and by thermodilution averaged over three consecutive measurements for healthy controls and patients with PCPH. CO was indexed relative to body surface area (BSA) as cardiac index (CI). Mean arterial blood pressure (MAP) and heart rate (HR) were measured non-invasively. Stroke volume index (SVI = CI/HR), PVR ([mPAP-PAWP]/CO), pulmonary arterial compliance (PAC = SV/pulmonary arterial pulse pressure), and RV stroke work index (RVSWI = SVI × [mPAP-RAP] × 0.0136) were calculated [15, 16].

2D echocardiograms were attained with a 3.5 MHz transducer on a GE vivid E95 (GE Healthcare, USA). Echocardiography was performed immediately following the invasive measurements. All images were stored digitally and analyzed post-hoc with EchoPAC software (General Electric Vingmed Ultrasound, USA). The echocardiographic measures were averaged over 3 consecutive beats at sinus rhythm or 4–6 beats at atrial fibrillation. RV parameters were calculated according to guidelines from an RV-focused apical view [17]. RV GLS, free wall strain (FWS), tricuspid annular peak systolic velocity (RV S’), tricuspid annular plane systolic excursion (TAPSE), and RV fractional area change (RV FAC) were measured [17]. GLS measures are reported as absolute values. Pulmonary arterial systolic pressure (PASP) was assessed by applying the Bernoulli equation to the tricuspid regurgitant jet peak velocity, and RAP was added. The TAPSE/PASP-ratio was calculated [18]. LVEF was calculated by Simpson biplane method and GLS was calculated from LV-focused apical views using a 17-segment model.

**Fig. 1:**
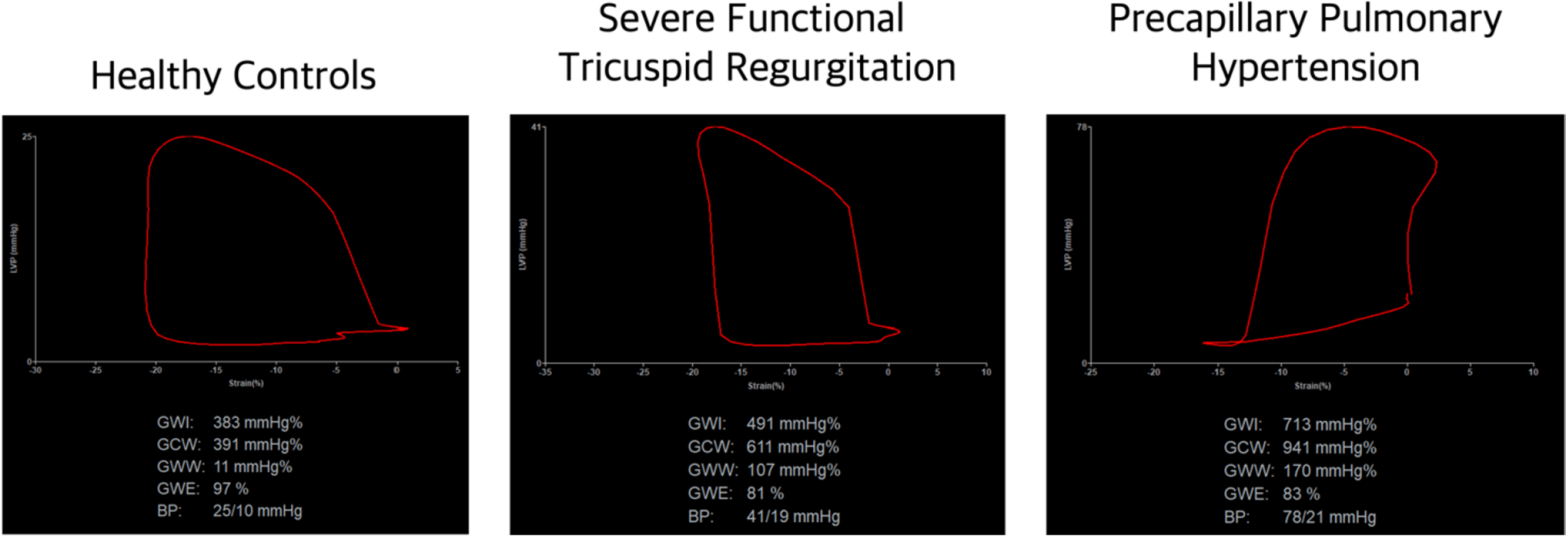
Representative pressure-strain loops from healthy controls and patients with severe functional tricuspid regurgitation or precapillary pulmonary hypertension

The novel indices of RV myocardial work were analyzed using specialized software designed to acquire LV myocardial work by combining two-dimensional speckle tracking echocardiography and blood pressure (EchoPAC Version 204). This enabled the synchronization of RV GLS values with simultaneous invasively measured pulmonary pressures. First, RV GLS was attained, followed by cardiac cycle timing with the pulmonic (through a pulsed-wave doppler in the RV outflow tract) and tricuspid valves (through visual rectification) combined with pulmonary pressures; RV pressure-strain loops were constructed within the software (Fig. 1). Four parameters were attained: (1) RV global work index (RVGWI; mmHg%), (2) RV global constructive work (RVGCW; mmHg%), (3) RV global wasted work (RVGWW; mmHg%), and (4) RV global work efficiency (RVGWE; %). RVGWI describes the area calculated from tricuspid valve closure to opening within the global RV pressure-strain loop; RVGCW describes the work needed for systolic myocardial shortening and isovolumic relaxation; RVGWW describes the wasted work during each cardiac cycle (i.e., myocyte lengthening during systole and shortening during isovolumic relaxation); and RVGWE describes the relation between RVGCW and the sum of RVGCW and RVGWW.

Continuous data are presented as mean and standard deviation or median and interquartile range, while categorical variables are presented as number and frequency. Normality distribution was assessed by QQ-plots and histograms. For comparisons among groups, continuous data were compared using the one-way ANOVA test for normal distributed data, and the Kruskal-Wallis test for non-parametric data. The chi-squared test was performed for categorical data. Pearson’s coefficient was used to assess correlation between continuous variables. Inter- and intraobserver agreement of RVMW in patients with PCPH (n = 9) or FTR (n = 10) were assessed by Bland-Altman plots and intraclass correlation coefficients (ICC). Thus, patient exams were selected in random to assess the reproducibility of RVMW. A two-sided *P*-value < 0.05 was considered statistically significant. Statistical tests were performed using STATA version 16 (StataCorp LP, Texas). Figures were created in GraphPad Prism 9.2 (GraphPad Software, California).

## Results

Patient characteristics are presented in Table 1. Age differed between the groups; healthy controls were younger (*P* < 0.001) and patients with FTR were older than patients with PCPH (*P* < 0.001). Comorbidities were equally distributed between patients with FTR and PCPH, except for an increased prevalence of hypertension in patients with FTR (*P* = 0.01). Patients with FTR received more frequently antihypertensive and anticoagulant medication, in addition with diuretics. Meanwhile, patients with PCPH received guideline recommended medication as appropriate (Supplementary Table 1). Finally, N-terminal pro brain-natriuretic-peptide (NT-proBNP) was significantly higher among patients with FTR as compared with PCPH patients (*P* < 0.001). Five patients with FTR (23%) and 2 patients with PCPH (10%) had RBBB.

**Table 1:** Baseline characteristics

	Healthy controls (n = 17)	FTR (n = 22)	PCPH (n = 20)	*P-*value
Age, years	49 ± 13	78 ± 5^†^	59 ± 17^–†‡^	< 0.001
Male Sex	12 (71%)	11 (50%)	5 (25%)^†‡^	0.021
BMI, kg/m^2^	24 ± 2	28 ± 5^†^	26 ± 5	0.013
NYHA/WHO-class I/II/III, n	–	1/10/11	3/14/3	0.046
Comorbidities				
Hypertension	–	13 (59%)	4 (20%)	0.010
Dyslipidemia	–	7 (32%)	3 (15%)	0.201
Diabetes Mellitus	–	4 (18%)	0	0.063
Ischemic Heart Disease	–	2 (9%)	0 (0%)	0.167
CKD (eGFR < 60 mL/min/1.73 m^2^)	–	8 (36%)	6 (30%)	0.662
Medication				
ACEi or ARB	–	10 (45%)	3 (15%)	0.033
Betablockers	–	12 (55%)	0 (0%)	< 0.001
Loop Diuretics	–	17 (77%)	9 (45%)	0.031
Mineralocorticoid receptor antagonist	–	7 (32%)	1 (5%)	0.027
Oral Anticoagulation	–	21 (95%)	13 (65%)	0.012
Calcium channel blockers	–	8 (38%)	1 (6%)	0.020
Laboratory data				
NT-proBNP, pmol/L	37 (34, 96)	1245 (817, 1849) ^†^	466 (132, 635) ^†‡^	< 0.001
eGFR, mL/min/1.73 m^2^	92 ± 11	68 ± 20^†^	79 ± 28	0.006
Hemoglobin, mmol/L	9.0 ± 0.9	8.4 ± 0.9	9.0 ± 0.9	0.063

Values are n (%), mean ± SD or median (interquartile range)

*ACEi* angiotensin-converting enzyme inhibitor, *ARB* angiotensin receptor blocker, *CKD* chronic kidney disease, *eGFR* estimated glomerular filtration rate, *FTR* functional tricuspid regurgitation, *NT-proBNP* N-terminal pro brain-natriuretic-peptide, *NYHA* New York Heart Association, *PCPH* precapillary pulmonary hypertension, *WHO* World Health Organization

^†^Significantly different versus controls

^†‡^Significantly different versus FTR

The echocardiographic parameters are presented in Table 2. RV GLS was reduced in both patient categories as compared with healthy controls (*P* < 0.001), however, there was no significant difference between patients with FTR and PCPH. Meanwhile, RV FWS, TAPSE, and TAPSE/PASP-ratio differed between all three study groups: these were highest in healthy controls, lower in patients with FTR, and lowest among patients with PCPH (*P* < 0.001 for all parameters). LVEF was significantly lower in patients with FTR compared with controls, while no significant difference in LV GLS was observed. LV hypertrophy was more pronounced in patients with FTR compared with the other study groups (*P* < 0.001).

**Table 2:** Echocardiographic parameters

	Healthy controls (n = 17)	FTR (n = 22)	PCPH (n = 20)	*P-*value
RVGWI, mmHg%	417 ± 73	508 ± 179	646 ± 242^†‡^	0.003
RVGCW, mmHg%	454 ± 73	687 ± 203^†^	881 ± 255^†‡^	< 0.001
RVGWW, mmHg%	10 (6, 17)	91 (53, 140)^†^	110 (66, 159)^†^	< 0.001
RVGWE, %	96 ± 3	86 ± 8^†^	86 ± 10^†^	< 0.001
TAPSE/PASP, mm/mmHg*	1.34 ± 0.39	0.78 ± 0.35^†^	0.43 ± 0.32^†‡^	< 0.001
RV GLS, %	23.7 ± 3.2	16.7 ± 4.0^†^	15.9 ± 3.4^†^	< 0.001
RV FWS, %	27.8 ± 3.9	21.8 ± 4.8^†^	16.5 ± 4.6^†‡^	< 0.001
RV S’, cm/sec	11.6 ± 1.6	11.2 ± 1.7	11.7 ± 3.5	0.78
TAPSE, mm	27 ± 3	21 ± 4^†^	16 ± 4^†‡^	< 0.001
RV FAC, %	46.2 ± 6.2	37.9 ± 6.4^†^	29.5 ± 11.4^†‡^	< 0.001
RV Basal Diameter, mm	35 ± 2	51 ± 7^†^	47 ± 6^†^	< 0.001
RV Mid Diameter, mm	27 ± 3	42 ± 10^†^	38 ± 7^†^	< 0.001
LVEF, %	63 ± 5	59 ± 5^†^	58 ± 7^†^	0.021
LV GLS, %	20 ± 2	17 ± 3	18 ± 3	0.051
LV Mass Index, g/m^2^	77 ± 14	96 ± 24†	71 ± 22^‡^	< 0.001

Values are mean ± SD or median (interquartile range)

*LVEF* left ventricular (LV) ejection fraction, *LV GLS* LV global longitudinal strain, *PASP* pulmonary artery systolic pressure, *RV FAC* RV fractional area change, *RV FWS* RV free wall longitudinal strain, *RV GLS* RV global longitudinal strain, *RVGCW and RVGWW* RV global constructive and wasted work, *RVGWE and RVGWI* RV global work efficiency and index, *RV S’* tricuspid annular peak systolic velocity, *TAPSE* tricuspid annular plane systolic excursion. Other abbreviations as in Table 1

*Available in 44/59 (75%) echocardiographic exams

^†^Significantly different versus controls

^†‡^Significantly different versus FTR

**Fig. 2:**
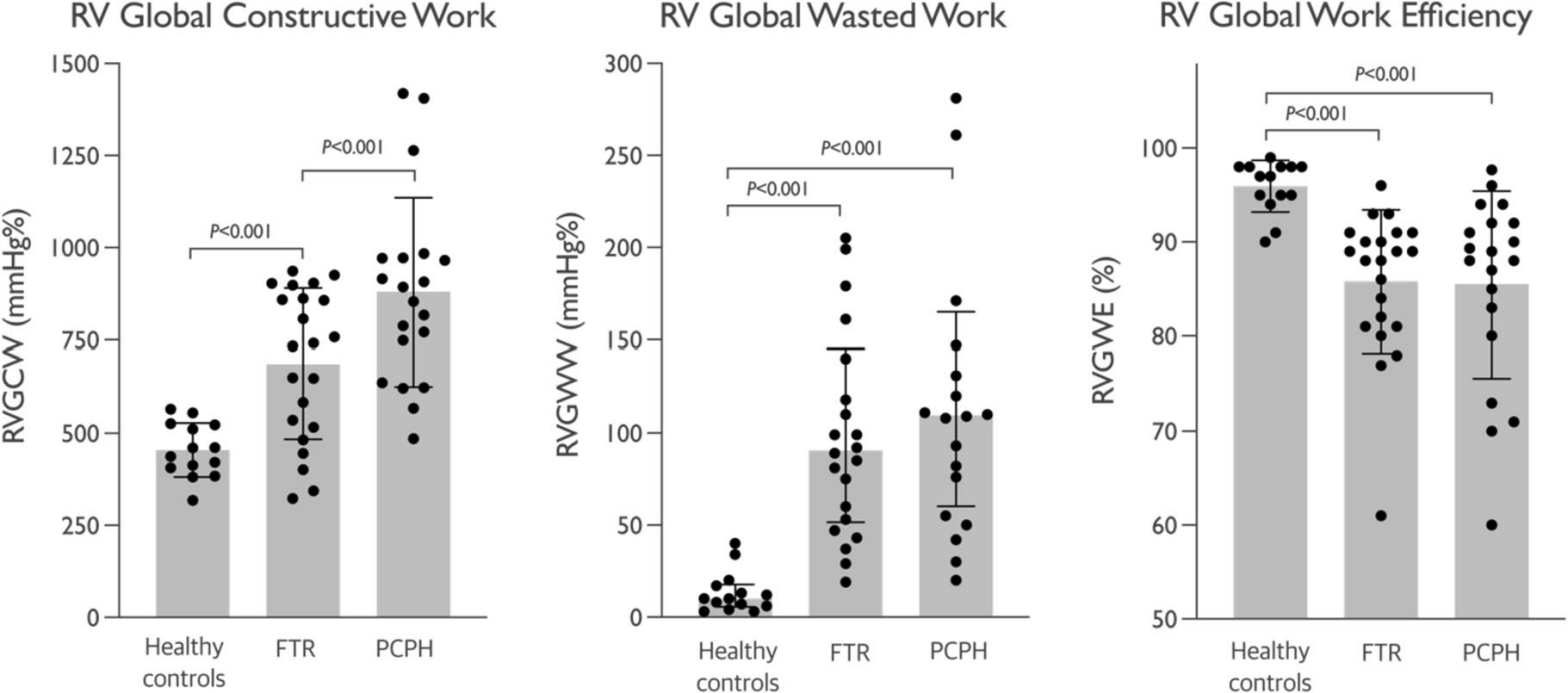
RV global constrictive, wasted, and work efficiency in patients with FTR or PCPH and healthy controls

RVMW parameters derived from pressure-strain calculations are displayed in Table 2; Fig. 2. RVGWI (*P* = 0.02) and RVGCW (*P* = 0.003) were significantly higher in patients with PCPH compared with FTR patients. RVGCW and RVGWW were greater in both patient groups as compared with controls (*P* < 0.001 for both values). In parallel, RVGWE was reduced in both patient groups compared with controls (*P* < 0.001). After adjusting for age, sex, and body mass index (BMI) RVGWE remained significantly reduced in patients with PCPH (7.9 ± 3.2%, *P* = 0.02), but not in patients with FTR (3.5 ± 3.9%, *P* = 0.37) as compared with controls. RVGWE was independently influenced by age; for every 1-year increment in age RVGWE decreased by 0.19 ± 0.8 (*P* = 0.02) percentage points (Supplementary Table 2).

There was no significant difference in CI, SVI, and SVO_2_ between patients with FTR or PCPH, whereas mPAP, PVR, and RVSWI were higher in the latter study group (*P* < 0.001 for all parameters; Table 3). RAP and PAWP were elevated in patients with FTR compared with patients with PCPH (*P* < 0.001).

**Table 3:** Right heart catheterization parameters

	Healthy controls (n = 17)	FTR (n = 22)	PCPH (n = 20)	*P-*value
Heart Rate, min^−1^	63 ± 9	74 ± 14^†^	70 ± 10	0.033
MAP, mmHg	92 ± 10	101 ± 16	93 ± 12	0.080
CI, L/min/m^2^	3.1 ± 0.6	2.2 ± 0.5^†^	2.3 ± 0.4^†^	< 0.001
SVI, mL/m^2^	48.8 ± 9.5	25.9 ± 7.6^†^	34.1 ± 6.7^†‡^	< 0.001
RVSWI, g m/m^2^/beat	6.8 ± 1.5	5.7 ± 2.9	16.4 ± 6.1^†‡^	< 0.001
RAP, mmHg	5 ± 2	12 ± 5^†^	5 ± 4^‡^	< 0.001
mPAP, mmHg	15 ± 2	28 ± 8^†^	39 ± 10^†‡^	< 0.001
PCWP, mmHg	9 ± 2	17 ± 5^†^	10 ± 4^‡^	< 0.001
PVR, WU	1.2 ± 0.3	2.7 ± 1.2^†^	7.1 ± 2.4^†‡^	< 0.001
PAC, mL/mmHg	8.5 ± 2.4	2.2 ± 1.1^†^	1.8 ± 0.8^†^	< 0.001
SVO_2_, %	76 ± 4	65 ± 5^†^	66 ± 5^†^	< 0.001

Values are mean ± SD or median (interquartile range)

*CI* cardiac index, *MAP* mean arterial pressure, *mPAP* mean pulmonary arterial pressure, *PAC* pulmonary artery compliance, *PCWP* pulmonary capillary wedge pressure, *PVR* pulmonary vascular resistance, *RAP* right atrial pressure, *RVSWI* right ventricular stroke work index, *SVI* stroke volume index, *SVO*_2_ mixed venous oxygen saturation. Other abbreviations as in Table 1

^†^Significantly different versus controls

^†‡^Significantly different versus FTR

**Fig. 3:**
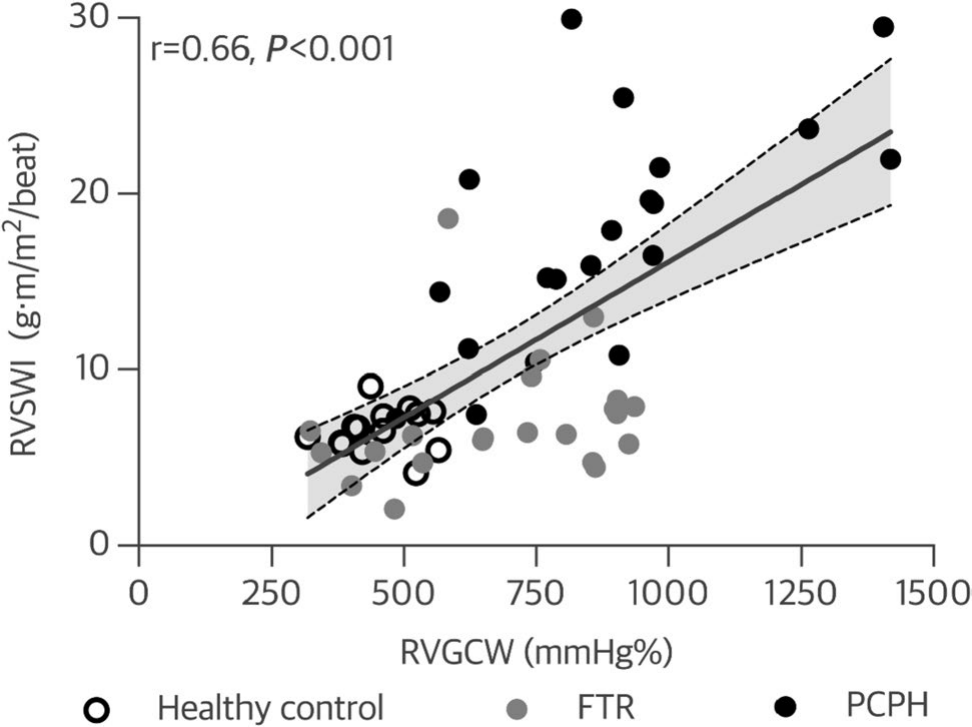
Correlation between RV global constructive work and RV stroke work index

The novel method of RV pressure-strain loops was compared to an invasively measured index of right ventriculoarterial coupling, namely RVSWI. In a pooled analysis, there was a good correlation between RVGCW and RVSWI (r = 0.66, *P* < 0.001; Fig. 3), however this was only confirmed in patients with PCPH (r = 0.62, *P* = 0.004; Table 4). Meanwhile, there was no significant correlation between RVGCW and RVSWI in controls (r = 0.36, *P* = 0.20) or patients with FTR (r = 0.11, *P* = 0.62; Table 4). RVGCW was highly dependent on RV afterload and correlated significantly with mPAP (r = 0.80, *P* < 0.001), PVR (0.74, *P* < 0.001), and PAC (− 0.63, *P* < 0.001; Supplementary Fig. 1). In patients with PCPH, RV FWS showed mild correlation with RVSWI, while no correlation was apparent in the other study groups (Table 4). Other echocardiographic parameters did not correlate significantly with RVSWI (Table 4).

**Table 4:** Correlation between echocardiographic parameters and right ventricular stroke work index within each study group

RVSWI (g m/m^2^/beat)	Healthy controls (n = 17)			FTR (n = 22)			PCPH (n = 20)		
	*ρ*	ß (SE)	*P* value	*ρ*	ß (SE)	*P* value	*ρ*	ß (SE)	*P* value
RVGCW, mmHg%	0.36	17.7 (13.0)	0.20	0.11	7.9 (15.9)	0.62	0.62	25.8 (7.8)	0.004
TAPSE/PASP, mm/mmHg	− 0.32	− 0.1 (0.1)	0.34	− 0.1	− 0.01 (0.01)	0.71	− 0.29	− 0.02 (0.02)	0.43
TAPSE, mm	0.41	0.9 (0.5)	0.12	0.17	0.3 (0.3)	0.45	0.33	0.2 (0.1)	0.15
RV FWS, %	− 0.29	− 17.4 (16.8)	0.32	− 0.08	− 7.0 (20.9)	0.74	− 0.55	− 24.6 (9.2)	0.02
RV GLS, %	− 0.20	− 0.4 (0.6)	0.49	0.20	0.3 (0.3)	0.43	− 0.13	− 0.1 (0.1)	0.58
RV S’, cm/sec	N/A	N/A	N/A	− 0.11	− 0.1 (0.1)	0.65	0.08	0.1 (0.1)	0.76

Abbreviations as in Table 2

RVGCW (r = 0.47, *P* = 0.04) and RVGWI (r = 0.66, *P* = 0.002) correlated with SVI in patients with PCPH (Supplementary Fig. 2), with no apparent correlation in the other study groups. However, this was not directly related to increased afterload, as there was no significant correlation between SVI and indices of RV afterload, i.e., mPAP (r = 0.27, *P* = 0.26) and PVR (0.15, *P* = 0.52) in patients with PCPH. We found no correlation between PVR and RVGWE nor RVGWW.

Bland-Altman plots for the assessment of inter- and intraobserver variability in patients with FTR and PCPH are shown in Supplementary Fig. 3. In patients with FTR, ICC for interobserver variability was 0.84 (95%CI 0.30–0.96) for RVGWI (*P* = 0.008), 0.88 (95%CI 0.54–0.97) for RVGCW (*P* = 0.002), 0.83 (95%CI 0.35–0.96) for RVGWW (*P* = 0.005), and 0.92 (95%CI 0.71–0.98) for RVGWE (*P* < 0.001); ICC for intraobserver variability was 0.87 (95%CI 0.50–0.97) for RVGWI (*P* = 0.002), 0.95 (95%CI 0.80–0.99) for RVGCW (*P* < 0.001), 0.95 (95%CI 0.80–0.99) for RVGWW (*P* < 0.001), and 0.88 (95%CI 0.56–0.88) for RVGWE (*P* = 0.001; Supplementary Table 3). In patients with PCPH, ICC for interobserver variability was 0.87 (95%CI 0.39–0.97) for RVGWI (*P* = 0.006), 0.81 (95%CI 0.23–0.96) for RVGCW (*P* = 0.01), 0.71 (95%CI − 0.21–0.93) for RVGWW (*P* = 0.04), and 0.81 (95%CI 0.20–0.96) for RVGWE (*P* = 0.01); ICC for intraobserver variability was 0.90 (95%CI 0.59–0.98) for RVGWI (*P* = 0.001), 0.92 (95%CI 0.61–0.98) for RVGCW (*P* = 0.002), 0.50 (95%CI –0.58–0.88) for RVGWW (*P* = 0.13), and 0.88 (95%CI 0.49–0.97) for RVGWE (*P* = 0.003; Supplementary Table 3).

## Discussion

In this study, we investigated RV function by integrating strain and pulmonary pressure to assess RVMW by pressure-strain loops in patients with RV hemodynamic overload and healthy controls. The main findings were: (1) RVGCW increased in tandem with higher afterload, i.e., was low among healthy controls, moderate in patients with FTR (i.e., RV volume overload), and highest among patients with PCPH (i.e., RV pressure overload); (2) RVGCW was highly dependent on RV afterload and correlated well with invasively measured RVSWI among patients with PCPH, but not in patients with FTR; (3) RVGWE was lower and RVGWW was higher among patients with FTR or PCPH compared to healthy control subjects, however, in patients with FTR this was partly explained by older age; (4) There was modest to good inter- and intraobserver repeatability of most RVMW parameters in patients with FTR and PCPH.

The RV possesses a unique ability to adapt to immense chronic increases in afterload by augmenting contractility to preserve cardiac output [19]. Therefore, both contractility and afterload (i.e., right ventriculoarterial coupling) must be assessed to evaluate RV dysfunction. RVSWI reflects the area inside the RV pressure-volume loop and comprises ventricular stroke volume in combination with preload and afterload. It is closely correlated with myocardial oxygen consumption and reflects the ventricular work to produce hydraulic energy, generating forward stroke work [20]. Several studies on patients with PCPH report decreased RVSWI to be independently associated with increased mortality [21, 22]. These findings reflect the transition from clinically compensated homeometric RV adaptation (i.e., increased contractility to match augmented afterload) to decompensated heterometric maladaptation (i.e., contractility is unable to match afterload, resulting in “ventriculoarterial uncoupling”) [23]. We observed a good correlation between RVSWI and echocardiographic-derived RVGCW in patients with PCPH, but not in patients with FTR. This may be explained by the regurgitant volume from the RV to the right atrium that contributes to the overall RV stroke work in patients with FTR. Consequently, this is better captured by RVGCW rather than RVSWI alone which is closely linked to forward stroke volume. Thus, by implementing valvular event-timings, RVGCW closely reflects RV stroke work, disregarding ventricular dyssynchrony and post-systolic contraction as wasted work. Distinct values of RVGCW were demonstrated within each study group. Evaluating RVGCW may thus aid to assess RV dysfunction in different right heart pathologies. Furthermore, RVGCW may serve as an integrative index of right ventriculoarterial coupling to monitor treatment response and disease progression in patients with RV hemodynamic overload. There was an apparent correlation between RVGCW and indices of RV afterload. As the patients with PCPH were stable, this finding may reflect preserved right ventriculoarterial coupling in these patients, i.e., homeometric RV adaptation to chronic pressure overload. Indeed, RVGCW was found to correlate with stroke volume in these patients. Hence, myocardial function (as measured with longitudinal strain) was sufficient to match the increase in afterload, and so, echocardiographic pressure-strain measurements associated with invasively measured pressure-flow measurements (i.e., RVSWI). Meanwhile, patients with FTR had higher RV filling pressures and increased indices of RV end-diastolic volume, in addition to increased FAC, all of which may indicate heterometric maladaptation [2]. Stroke volume and RVSWI were reduced, while RVGCW remained increased (relative to healthy controls), indicating increased RV work in the presence of increased backward flow. Therefore, monitoring RVGCW may aid in assessing progression of RV dysfunction in patients with FTR.

RV mechanical dyssynchrony comprises an important pathophysiological feature in PCPH [6]. Delayed time-to-peak contraction of the RV is evident in PAH [24, 25], causing mechanically inefficient post-systolic isovolumetric contraction (i.e., wasted contractile work) [26]. In agreement with the literature, we discovered an increase in RV wasted work in both patient categories of RV hemodynamic overload. Consequently, RVGWE was impaired, consistent with RV mechanical inefficiency due to chronic hemodynamic overload. As RVGWE is dependent on the RV constructive-to-wasted work ratio, it reflects the mechanical determinants of this relationship, especially, forward stroke work and inefficient post-systolic shortening. By accounting for and quantifying the magnitude of the prolonged post-systolic period, RVGWW may reflect the degree of RV electromechanical dyssynchrony. Indeed, the degree of RV dyssynchrony correlates with the severity of PCPH, distinguishing healthy controls from borderline and manifest PAH [6, 19, 27]. Thus, impaired RVGWE during follow-up of patients with PCPH may reflect disease progression including RV dysfunction due to either increased wasted work, reduced constructive work, or both combined. Echocardiographic assessment of RV dysfunction remains challenging due to the intrinsic right ventriculoarterial relationship and complex ventricular anatomy. TAPSE and S’ are well-validated and easily obtained measures of RV systolic function. However, they are dependent on cardiac angle and motion relative to the ultrasound transducer [28]. Next, RV longitudinal strain is a reproducible measure of RV systolic function and shows prognostic value in patients with PAH or FTR [29, 30]. Yet, it is load dependent, limiting the evaluation of PAH [31]. Validation of three-dimensional echocardiographic RV ejection fraction versus CMR has shown promising results [32] but is hampered by imperfect image quality, load dependency, possible severe TR, and interventricular dyssynchrony causing septal bouncing [17, 18]. TAPSE/PASP-ratio has been utilized to evaluate right ventriculoarterial coupling in patients with severe FTR, demonstrating prognostic information [18]. Nevertheless, the TAPSE/PASP-ratio is limited by the abovementioned limitations. In this regard, we found no correlation between TAPSE/PASP-ratio and RVSWI in patients with FTR or PCPH. By incorporating both contractile function and afterload, RVMW provides a less load dependent index of ventricular performance. We discovered highly comparable values of RVGWI, RVGCW, RVGWW, and RVGWE in healthy controls and patients with PCPH as previously reported [9, 10]. Furthermore, inter- and intraobserver variability was modest to good except for RVGWW, which may be limited by imperfect valvular event timing and small values. In the present study, we provide information that extends the generalizability of RVMW to patients with various forms of RV hemodynamic overload, including RV pressure and volume overload. Importantly, these measures can be easily incorporated into clinical practice, as they require minimal extra acquisition time and demonstrate good reproducibility, including consistent results across different laboratories [10].

Due to the cross-sectional design of this study, potential limitations should be considered. First, disease duration remains unknown and cannot be accounted for. Second, despite an association between RVGCW and RVSWI, correlation does not necessitate causation. Third, because of the retrospective nature of this investigation, study groups were heterogeneous with respect to baseline characteristics, including differences in age, sex distribution, and BMI. Nevertheless, each study group was internally homogenous with respect to baseline characteristics and hemodynamic profiles and the healthy control demonstrated no signs of RV volume overload. Moreover, our results align with those reported by Butcher et al. [10], and reinforces the external validity and reproducibility of echocardiographic indices of RV myocardial work. The present data are based on combining echocardiographic RV longitudinal strain and invasive measures of pulmonary pressures. Hence, RHC was presupposed. Whether non-invasive derived pulmonary pressures would produce similar results remain undetermined. However, this may only be feasible in selected patients in whom TR flow velocity can be measured validly without a laminar flow profile. Another limitation is that RVMW was evaluated using software originally created and tested for evaluation of LVMW [8]. Patients with FTR had chronic atrial fibrillation. As the evaluation of RVMW requires valvular event timings, variation in cardiac cycle length may pose a potential limitation. Still, we observed good interobserver agreement in the RVMW measurements in these patients. Moreover, studying FTR in patients with atrial fibrillation is highly relevant, as these patients represent the majority of patients with FTR [33]. Finally, LV pressure-strain loops are validated by invasive pressure-volume loops, using brachial artery blood pressure and simple geometric assumptions [8]. The irregular RV geometry challenges these assumptions, making volumetric assessment difficult [2]. Despite this, RV pressure-strain loops rely less on volume by measuring longitudinal force-segment changes. Future trials are crucial for exploring the correlation between invasive RV pressure-volume and pressure-strain loops using echocardiography.

In conclusion, assessment of RVMW is feasible in patients with RV hemodynamic overload and differs from healthy controls. RVGCW correlates well with invasive assessment of RVSWI in patients with RV pressure overload but not in patients with RV volume overload. Furthermore, this method enables quantification of RVGWW and RVGWE. Whether this novel method possesses prognostic value, and whether it can be applied to assess specific treatment effects and disease progression needs to be determined in long-term future studies.

### Supplementary Information

Below is the link to the electronic supplementary material.

Supplementary material 1 (DOCX 466.0 kb)
