# Supplementary material for: Myocardial work across different etiologies of right ventricular dysfunction and healthy controls
Source: Int J Cardiovasc Imaging. 2024 Feb 2;40(3):675–84. doi: 10.1007/s10554-023-03038-y (PMC10950966; doi:10.1007/s10554-023-03038-y)
Supplement: Supplementary file 1 — Supplementary material 1 (DOCX 466.0 kb) [file 10554_2023_3038_MOESM1_ESM.docx]

**Myocardial Work across different Etiologies of Right Ventricular Dysfunction and Healthy Controls**

**Supplementary Material**

# Supplementary Figure 1: Correlation between RVGCW and indices of RV afterload

There was good correlation between right ventricular (RV) global constructive work (RVGCW) and indices of RV afterload, i.e., **A)** mean pulmonary arterial pressure (mPAP); **B)** pulmonary vascular resistance (PVR); and **C)** pulmonary artery compliance (PAC). FTR = functional tricuspid regurgitation; PCPH = precapillary pulmonary hypertension.

# Supplementary Figure 2: Correlation between SVI and RVGCW and RVGWI

There was significant correlation between RV stroke volume index (SVI) and RVGCW and RV global work index (RVGWI) in patients with PCPH but not in patients with FTR or healthy controls. The panel to the left (**A, C, E**) displays the correlation between SVI and RVGCW. The panel to the right (**B, D, F**) displays the correlation between SVI and RVGWI. **A, B**) Healthy controls; **C, D**) FTR; **E, F**) PCPH. Abbreviations as in Supplementary Figure 1.

**Supplementary Figure 3:** Bland-Altman plots for inter- and intraobserver variability for RV myocardial work in patients with FTR and PCPH

RVGWW = RV global wasted work; RVGWE and RVGWI = RV global work efficiency and index. Upper and lower grid lines indicate 95% limits of agreement. Abbreviations as in Supplementary Figure 1.

**Supplementary Table 1:** Baseline specific treatment of patients with precapillary pulmonary hypertension

| **Treatment** | **N = 20** |
| --- | --- |
| PDE5i | 14 (82%) |
| ERA | 10 (50%) |
| PGa | 3 (16%) |
| sGCS | 2 (12%) |
| BPA | 7 (35%) |
| PTEA | 8 (40%) |

Values are n (%). BPA = Balloon pulmonary angioplasty; ERA = Endothelin receptor antagonists; PDE5i = Phosphordiesterase-5 inhibitors; PGa = Parental prostaglandin analogue; PEA = Pulmonary endarterectomy; PCPH = Precapillary Pulmonary Hypertension; sGCS = soluble guanylate cyclase inhibitors

**Supplementary Table 2:** Multivariate model showing the influence of sex, age, body mass index, and study group on right ventricular global work efficiency.

|  | **ß (SE)** | ***P*-value** |
| --- | --- | --- |
| Male sex | 0.15 (2.33) | 0.95 |
| Age, y | -0.19 (0.08) | 0.02 |
| BMI, kg/m^2^ | -0.23 (0.24) | 0.24 |
| Study group: Healthy controls as reference group | | |
| FTR | -3.50 (3.89) | 0.37 |
| PCPH | -7.93 (3.23) | 0.02 |

BMI = body mass index; FTR = functional tricuspid regurgitation; PCPH = precapillary pulmonary hypertension

**Supplementary Table 3:** Inter- and intraobserver variability for RVMW in patients with FTR and PCPH

|  | **Interobserver variability** | | **Intraobserver variability** | |
| --- | --- | --- | --- | --- |
|  | ICC (95% CI) | *P* value | ICC (95% CI) | *P* value |
| **Patients with FTR (n = 10)** | | | | |
| RVGWI | 0.84 (0.30-0.96) | 0.008 | 0.87 (0.50-0.97) | 0.002 |
| RVGCW | 0.88 (0.54-0.97) | 0.002 | 0.95 (0.80-0.99) | <0.001 |
| RVGWW | 0.83 (0.35-0.96) | 0.005 | 0.95 (0.80-0.99) | <0.001 |
| RVGWE | 0.92 (0.71-0.98) | <0.001 | 0.88 (0.56-0.88) | 0.001 |
| **Patients with PCPH (n = 9)** | | | | |
| RVGWI | 0.87 (0.39-0.97) | 0.006 | 0.90 (0.59-0.98) | 0.001 |
| RVGCW | 0.81 (0.23-0.96) | 0.01 | 0.92 (0.61-0.98) | 0.002 |
| RVGWW | 0.71 (-0.21-0.93) | 0.04 | 0.50 (-0.58-0.88) | 0.13 |
| RVGWE | 0.81 (0.20-0.96) | 0.01 | 0.88 (0.49-0.97) | 0.003 |

RVGCW and RVGWW = RV global constructive and wasted work; RVGWE and RVGWI = RV global work efficiency and index. Abbreviations as in Supplementary Figure 2.
